# Diagnostic challenge of the newborn patients with heritable protein C deficiency

**DOI:** 10.1038/s41372-018-0262-0

**Published:** 2018-10-23

**Authors:** Masako Ichiyama, Hirosuke Inoue, Masayuki Ochiai, Masataka Ishimura, Akira Shiraishi, Junko Fujiyoshi, Hironori Yamashita, Kazuo Sato, Shinya Matsumoto, Taeko Hotta, Takeshi Uchiumi, Dongchon Kang, Shouichi Ohga

**Affiliations:** 1 Comprehensive Maternity and Perinatal Care Center, Kyushu University Hospital, Fukuoka, Japan; 2 Department of Pediatrics, Graduate School of Medical Sciences, Kyushu University, Fukuoka, Japan; 3 Department of Perinatal and Pediatric Medicine, Graduate School of Medical Sciences, Kyushu University, Fukuoka, Japan; 4 Division of Pediatrics, National Hospital Organization Kokura Medical Center, Fukuoka, Japan; 5 Division of Pediatrics, National Hospital Organization Kyushu Medical Center, Fukuoka, Japan; 6 Department of Clinical Chemistry and Laboratory Medicine, Kyushu University Hospital, Fukuoka, Japan

**Keywords:** Haematological diseases, Genetics research

## Abstract

**Objective:**

The diagnosis of neonatal-onset protein C (PC) deficiency is challenging. This study aimed to establish the neonatal screening of heritable PC deficiency in Japan.

**Study design:**

We determined the changes in plasma activity levels of PC and protein S (PS) in healthy neonates, and studied newborn patients with *PROC* mutation in the Japanese registry.

**Result:**

Physiological PC and PS levels increased with wide range. The PC/PS-activity ratios converged after birth. The PC/PS-activity ratios of 19 patients with biallelic mutations, but not, 9 with monoallelic mutation, were lower than those of 13 without mutation. The logistic regression analyses established a formula including two significant variables of PC activity (cut-off < 10%, odds ratio = 30.0) and PC/PS-activity ratio (cut-off < 0.35, odds ratio = 22.7), with 93% sensitivity and 44% specificity for determining patients with mutation(s).

**Conclusion:**

The PC/PS-activity ratio is an effective parameter for the genetic screening of neonatal-onset PC-deficiency in Japanese population.

## Introduction

Neonatal thrombosis has been increasingly diagnosed in developed countries. It is ascribed to the advances in neonatal intensive care, cardiovascular surgery, and imaging diagnosis [1, 2]. Thromboembolism uncommonly occurs in children compared to adults, because of the maturation and aging of hemostatic and cardiovascular systems [3]. However, until 20 years of age, the incidence of thromboembolic events is at the highest in neonates, and at the second highest in adolescents [4, 5]. The dynamic changes of circulation at birth contribute to the development of arterial and venous thromboses in neonates. On the other hand, the substantial risk of thrombotic events in newborn patients depends on the genetic predisposition and triggers such as asphyxia and infection. The effect size of thrombophilia genes in individuals might be augmented during the perinatal and adolescent periods according to age-associated conditions, compared to the adult patients with thromboembolism [6].

The genetic risk of venous thromboembolism is increased in subjects with protein C (PC), protein S (PS) and antithrombin (AT) deficiencies, and modestly increased in those with factor V G1691A (FVL) and prothrombin G20210A (FII) variants [7, 8]. The allele frequency of FVL or FII variant shapes the landscape of molecular epidemiology for inherited thrombosis in Caucasians [9–11]. By contrast, the absence of FVL or FII variant in East Asia raises the clinical impact of PC, PS, and AT deficiency on thromboembolism in Asian patients [12–14]. Severe PC deficiency is a rare thrombophilia. Infants with biallelic mutations in PC gene (*PROC*) develop purpura fulminans and/or hemorrhagic stroke within 5 days after birth [15]. Perinatal stresses can also predispose the heterozygotes for *PROC* mutation to fetal and neonatal thromboses [16–18]. The ongoing cohort in Japanese children since 2011 has reported that the patients with heterozygous mutation of *PROC* (*n* = 17, 59%) exceeded those with biallelic mutations (homozygous 3, compound heterozygous 9) in number among 29 pediatric patients who had *PROC* mutation(s) and thromboembolism [15–18]. However, the genetic test is an elaborative work for the early diagnosis and treatment of newborn patients with PC deficiency.

In the present study, to explore the rapid screening methods of neonatal-onset heritable PC deficiency, we first determined variable increase in plasma PC and PS activities and converging ratios of the PC/PS-activity in healthy infants after birth. By using the data of newborn patients in the Japanese registry, we have established a formula consisting of PC activity and PC/PS-activity ratio for discriminating the patients with *PROC* mutation from those without it. The population-specific approach for the diagnosis of neonatal-onset heritable PC deficiency is discussed for an extension of the personalized medicine in early life.

## Materials and methods

### Study subjects and patients

The control levels of plasma PC or PS activity after birth were determined by using the same samples obtained from 46 healthy infants (male: female, 21: 25; gestational age median 38 weeks, range: 37–41 weeks; birth weight median 3024 g, range: 2500–3810 g) born in our institutions between 2013 and 2015. The plasma activity of PC and PS was measured by the following method once in each subject within 28 days after birth. These infants had no any abnormality in the physical examination, complete blood counts, and the data of biochemistry and coagulation tests.

Forty-one infants who had thrombotic events within 28 days after birth and received the genetic tests were collected from the Japanese registry for pediatric PC deficiency. Twenty-three out of 38 patients had thrombotic events and received the genetic study for PC, PS and/or AT genes at Kyushu University from 1993 to 2016, and 18 of them received the diagnosis of PC deficiency from all publications and meeting reports from 1981 to 2016 [15–21]. The information was based on the clinical network consisting of 111 perinatal care centers in Japan from 2011 to 2016. The indication of genetic study was used for the established screening criteria of anticoagulant activity in childhood [22]. The collected data included gender, the age at the onset of each thromboembolic event, the family history of PC deficiency, PC antigen and activity levels, genetic study results, and the outcomes of patients. Both PC and PS activities were concurrently evaluated in 24 of 38 patients in the registry. Written informed consent was obtained from parents of the subjects and patients. This study was certified by the Institutional Review Board of Kyushu University (#448-00).

### Coagulation study

Coagulation tests were performed as described previously [22]. The anticoagulant activities of PC and PS were determined using the Staclot PC kit and the Staclot PS kit (Diagnostica Stago, Asnieres, France), respectively. A chromogenic substrate was used to assay for AT activity as heparin dependent inhibition of bovine thrombin (Chromostrate ATIII kit, Hitachi, Tokyo, Japan). The reference ranges of the activity and antigen levels of anticoagulants for neonates were based on the previous studies [22–26]. In the first study of healthy controls, protein induced by vitamin K absence or antagonists-II (PIVKA-II) was measured by the established method, irrespective of the administration of vitamin K2. In our cohort study of patients, the plasma PC activity was determined using repeated coagulation tests (including factor VII activity) at the time of diagnosis and during the disease course.

### Gene analysis of PC, PS, and AT

Genomic DNA was extracted from peripheral blood leukocytes after obtaining informed consent from the patients. The direct sequencing of polymerase chain reaction (PCR) products was performed for the coding regions of *PROC* (exons 1–9), PS (*PROS1* exon 1–15) and AT (*SERPINC1* exon 1–6) genes as described previously [18, 22]. The exon and exon–intron boundary regions of each gene, including the promoter region, were amplified by PCR, and the products were then subjected to direct sequencing using an ABI 377 (Applied Biosystems, Foster City, CA, USA).

### Statistical analysis

Univariate analyses were performed using Fisher’s exact test (dichotomous variables) and Wilcoxon rank-sum test (continuous variables). An analysis of covariance was used to compare the slope index of regression equations in PC and PS activities of healthy neonates. Spearman’s rank-sum test was used for the association study. For all analyses, a two-sided probability value below 0.05 was considered to indicate statistical significance. To identify the independent predictors for mutation carriers, univariate logistic regression models were constructed using demographic variables. Multivariate logistic regression analysis was performed using the stepwise logistic regression with backward elimination. The *p* value of <0.05 was required for entry into the model, and > 0.05 for elimination. Results were expressed as an odds ratio (OR) with a 95% confidence interval (CI). The discriminatory capacity of the model was assessed using the area under the receiver-operating-characteristics (ROC) curve. The goodness of fit of the regression model was tested with the Hosmer–Lemeshow test, with *p* > 0.05 considered to indicate lack of deviation between the model and observed event rate. All analyses were carried out using JMP Pro11 (version 11.2.0 for Windows; JMP Inc. SAS Institute Japan).

## Results

### PC and PS activity levels in healthy neonates

**Fig. 1:**
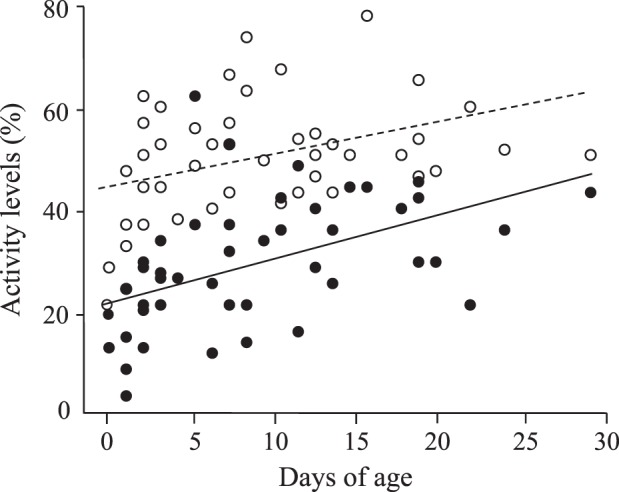
Plasma activity levels of protein C (PC) and protein S (PS) in healthy newborn infants. Both activity levels were measured by using 46 plasma samples concurrently procured from 46 subjects during the neonatal period. Open and closed circles represent PS and PC levels, respectively. Dashed and solid lines represent the linear regression line between the days after birth and PS levels (correlation coefficient [cc] = 0.40, *p* < 0.01) or PC levels (cc = 0.58, *p* < 0.01), respectively, assessed by the Spearman’s rank-sum test. The slope indices in the linear correlations did not differ between PC and PS activities assessed by the covariance analysis

**Fig. 2:**
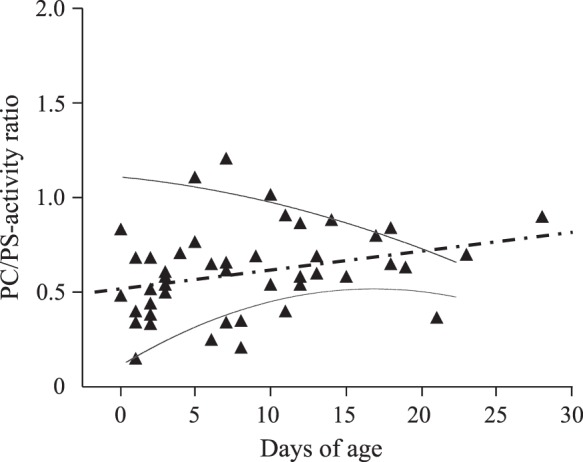
The plasma activity ratio of PC and PS in 46 healthy newborn infants. The dashed line represents the regression line between the days after birth and the PC/PS ratios. The positive correlation was significant showing the smaller gradient than 0. 4 (cc = 0.35, *p* < 0.05). The regression curves were calculated by the polynomial approximation with maximum and minimum levels

Plasma levels of PC and PS activities in the same samples of 46 healthy newborns during the first 1 month of life are illustrated in Fig. 1. The plasma levels of PC or PS activity showed the wide variation (median PC 31.0%, range: 5–68%; median PS 49.5%, range: 24–76%), irrespective of the vitamin K states (data not shown). Both PC and PS activity levels increased with days after birth (PC: correlation coefficient [cc] 0.58, *p* < 0.01, PS: cc 0.40, *p* < 0.01). Up to 14 days after birth, 8 infants showed <20% of PC but not PS activity. There was no difference in the increasing trends between PC and PS activity levels assessed by the covariance analysis of the gradient of regression line (*p* = 0.31). The PC/PS-activity ratios of the healthy newborns are shown in Fig. 2. The distribution of the ratios came to converge on the flat pattern (cc 0.35, *p* < 0.05) during the first 10 days of life (median = 0.61, range: 0.15–1.21).

### Clinical profile and PC/PS activities of the neonatal-onset PC deficienc*y*

The clinical and laboratory findings of 41 patients who received the clinical diagnosis of PC deficiency are shown in Table 1, according to the presence of biallelic mutations (2 homozygotes and 17 compound heterozygotes), monoallelic mutation, and nonmutation. The detailed information on the genotype of each patient is shown in Supplementary Table. When they were compared among those with and without *PROC* mutation(s), there was neither sex preponderance nor birth weight. The Apgar scores and gestational age in patients with mutation(s) were higher than seen in those without mutation (each *p* = 0.02, 0.04). The patients with mutation(s) or biallelic mutations showed a higher incidence of purpura fulminans (each *p* < 0.01, <0.01), or more than one manifestations (each *p* < 0.01,<0.01) than those without mutation. The PC activity levels and the PC/PS-activity ratios of patients with mutation(s) or biallelic mutations were lower than the PC levels and the PC/PS ratios of those without mutation, respectively (PC activity, each *p* < 0.01, <0.01, PC/PS ratio, each *p* < 0.01, <0.01, Table 1). On the other hand, only the onset of disease confirmed significant difference between monoallelic mutation and nonmutation. The phenotypes were similar in the homozygotes and compound heterozygotes for mutation. No PC levels differed between nonmutation patients and healthy infants <7 days after birth.

**Table 1:** Characteristics of infants with neonatal-onset protein C deficiency according to *PROC* gene mutation

	Mutation(s)^a^		Nonmutation	*p* Value		
	Biallelic	Monoallelic		a	b	c
	*n* = 19	*n* = 9	*n* = 13			
	No. (%)	No. (%)	No. (%)			
Sex male	10 (53)	3 (33)	5 (39)	>1	>1	>1
Gestational age > 36 weeks	17 (89)	9 (100)	8 (61)	0.02	0.08	0.05
Birth weight, grams, median, range	2815, 1854–3640	2901, 2560–3560	2469, 818–3305	0.15	0.40	0.06
Apgar score at 5 min, median, range	9, 6–10	9, 9–9	7, 4–9	0.02	0.04	0.43
*Onset of disease*						
Fetal	3 (18)^b^	1 (11)	1 (8)	>1	0.62	>1
0–6 days of age	13 (77)^b^	5 (56)	12 (92)	0.15	>1	0.02
7–28 days of age	1 (6)^b^	3 (33)	0 (0)	0.15	>1	0.02
*Clinical presentations*						
Intracranial thrombosis/hemorrhage	15 (79)	9 (100)	11 (84)	>1	>1	>1
Purpura fulminans	14 (74)	2 (22)	1 (8)	<0.01	<0.01	0.54
Ocular bleeding	6 (32)	1 (11)	0 (0)	0.08	0.06	0.41
Others^c^	1 (5)	0 (0)	1 (8)	>1	>1	>1
Number of manifestation > 1	13 (68)	2 (22)	1 (8)	<0.01	<0.01	0.54
Protein C (PC) activity, %, median, range	8, 2–24	17, 10–31	20, 6–45	<0.01	<0.01	0.13
Protein S (PS) activity, %, median, range	67, 24–140	47, 34–63	47, 36–62	0.76	0.59	0.80
PC activity/PS activity, median, range	0.11, 0.06–0.29	0.40, 0.27–0.50	0.49, 0.11–0.75	<0.01	<0.01	0.91

^a^Biallelic mutations mean homozygous or compound heterozygous mutation, and monoallelic mutation means heterozygous mutation

^b^Data are missing from 2 infants. a: mutation(s) vs. nonmutation, b: biallelic vs. nonmutation, c: monoallelic vs. nonmutation

^c^Other thrombotic presentations consisted of deep vein thrombosis (biallelic, *n* = 1) and intramuscular hematoma (nonmutation, *n* = 1)

*p* Values were obtained from Fisher's exact test (dichotomous variables) and Wilcoxon rank-sum test (continuous variables)

### Predictive factors for *PROC* mutation(s) in patients with neonatal-onset PC deficiency

**Fig. 3:**
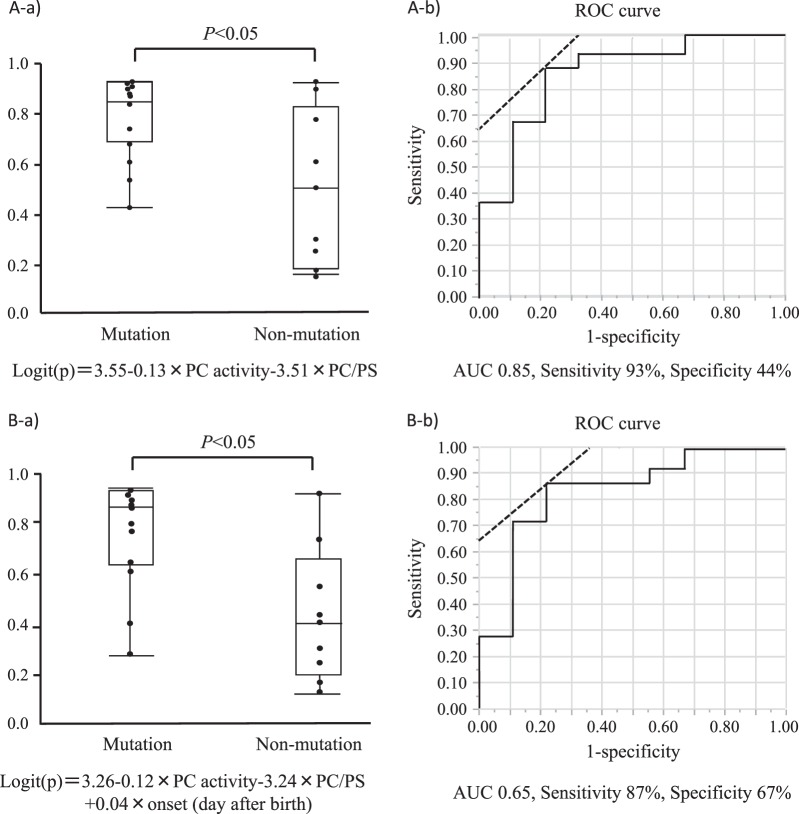
Predictive formulas for PROC mutation in infants with neonatal-onset PC deficiency. **a** The formula was drawn by the analysis on 24 infants having no defective variables (mutation: *n* = 15, nonmutation: *n* = 9) in the registry, consisting of 7 patients from publications and 17 patients analyzed in Kyushu University (panel a). The probability of mutation was calculated as follows; Logit(P) = 3.55-0.13 × PC activity-3.51 × PC/PS ratio. **b** The other formula was drawn by the analysis on 23 infants having no defective variables (mutation: *n* = 14, nonmutation: *n* = 9) in the registry, consisting of 7 patients from publications and 16 patients analyzed in Kyushu University (panel a). The probability of mutation was calculated as follows; Logit(P) = 3.26 − 0.12 × PC activity-3.24 × PC/PS + 0.04 × onset (day after birth). **a, b** The goodness of fit of the model was statistically significant assessed by the Hosmer–Lemeshow test (panel b)

A univariate analysis identified that six out of all variables were associated with the patients having biallelic or monoallelic mutation(s) in *PROC* (Table 2). The multivariate logistic regression analyses established two significant formulas for predicting *PROC* mutation(s) in neonatal thrombosis (Hosmer–Lemeshow statistics); (A) Logit (P) = 3.55 – 0.13 × PC activity – 3.51 × PC/PS ratio (Fig. 3a), and (B) Logit (P) = 3.26 – 0.12 × PC activity – 3.24 × PC/PS ratio + 0.04 × onset (days after birth) (Fig. 3b). The probability of model A was effectively discriminated the mutation(s)-carrying patients from nonmutation patients (median 0.84 vs. 0.52, *p* < 0.05). The area under the ROC curve was 0.85. For a cut-off point of 0.5, the sensitivity and specificity in the discrimination between mutation and nonmutation were 93% and 44%, respectively. Only one patient escaped the differentiation by formula A, who was a heterozygote of variant K193del as previously reported [27].

**Table 2:** Univariate analysis for variables associated with *PROC* mutation(s) in infants diagnosed with neonatal-onset PC deficiency

	Cut-off	Sensitivity (%)	Specificity (%)	PPV (%)	NPV (%)	AUC (95% CI)	Odds ratio (95% CI)	*p* Value
PC activity	<10%	71	92	95	60	0.82 (0.68–0.91)	30.0 (3.33–270)	<0.01
Purpura fulminans	yes	57	92	94	50	0.74 (0.61–0.85)	16.0 (1.82–140)	0.013
Manifestations	>1	54	92	94	48	0.73 (0.59–0.83)	13.9 (1.58–121)	0.017
Gestational age	>36 weeks	93	31	76	67	0.67 (0.50–0.80)	9.3 (1.47–58)	0.018
Apgar score at 5 min	>7	93	36	82	75	0.72 (0.45–0.89)	14.0 (1.06–185)	0.045
PC activity/PS activity	<0.35	87	78	87	78	0.82 (0.59–0.93)	22.7 (2.60–198)	<0.01

*PC* protein C, *PS* protein S, *PPV* positive predictive value, *NPV* negative predictive value, *AUC* the area under a receiver-operating characteristic curve, *CI* confidence interval. *p* Values were obtained from logistic regression analysis

## Discussion

We demonstrated that the physiological increase in PC and PS activity has a wide range of distributions during the first month of life, and that the ratio of PC to PS activity levels shows a less increasing slope with a narrower range than each activity level after birth. Not the PC/PS activity ratio alone, but a formula consisting of the PC/PS ratio and PC activity, effectively differentiated patients with heterozygous mutation from those without it. The PC/PS activity ratio is a useful parameter for the genetic screening of neonatal-onset PC deficiency.

Newborn infants exhibit the greatest risk for developing thrombosis in childhood [5]. Besides the maternal and placental factors, a variety of triggers in neonatal intensive cares affect the hemostatic balance toward thrombosis including invasive procedures of surgery or central venous catheterization, unstable fluid control, infection and inflammation. PC deficiency is the most common thrombophilia as the cause of pediatric thrombosis in Japan [28]. These patients have one allele or double allele mutations of *PROC*, and develop hemorrhagic infarction and/or purpura fulminans within two weeks after birth [14, 18], although thrombotic trend depends on the absolute PC activity levels [15]. Because of the varied hemostatic maturation [29], it is difficult to assess the balanced development of coagulation, anticoagulation and fibrinolysis factors during the early postnatal life. The levels of plasma PC activity reportedly show <20% in fetuses throughout the intrauterine life and a wide variation in normal full-term newborns (mean 28.2%, 5th–95th percentile range 14–42%) [30]. The postnatal values of plasma PC activity do not enter the adult reference range until puberty, that delineates the most delayed maturation after birth among the vitamin K-dependent factors. The present study revealed the distinct increasing pattern of PC activity from PS activity in healthy newborns. All healthy infants had >20% of PS activity levels, while 7 infants showed a moderate deficiency (1–20%) of PC activity within the first 10 days of life. There was no difference in the plasma levels of PC activity between healthy infants with and without vitamin K replacement (data not shown). Although these 7 infants with <20% of PC activity showed detectable levels of protein induced by PIVKA-II (median 123 μg/mL, range: 15–1562 μg/mL), their PS activity levels all exceeded 20% (Fig. 1). After 10 days of life, the PC/PS activity ratios came to converge on 0.6 ranging from 0.5 to 1.0 (Fig. 2). These results suggest that plasma PC activity levels are more sensitively controlled by vitamin K status than the PS activity levels until 10 days after birth. Alternatively, these may represent a different mechanism for the circulating levels of PC from PS during the early neonatal period.

Clinical presentation of adult patients with heterozygous PC deficiency varies from mild-to-severe thromboembolic diseases, according to the plasma levels of PC activity. By contrast, the neonatal-onset patients with biallelic mutations exclusively show <10% of plasma PC activity at diagnosis [15]. Of total 41 patients with neonatal-onset PC deficiency in the Japanese registry, 28 had *PROC* mutation(s), of whom 19 had biallelic mutations and 9 had heterozygous mutation. The infants with mutation(s) showed higher incidence of purpura fulminans, higher number of manifestations, and lower levels of PC activity and PC/PS-activity ratio than infants without mutation (Table 1). However, there was no significant difference in any variables between heterozygous mutation and nonmutation groups. These corroborate the clinical difficulty to distinguish between newborn patients with heterozygous *PROC* mutation and those without mutation. On the other hand, the gestational age and Apgar score in infants having no *PROC* mutation were lower than those in infants having no mutation (Table 1), which suggested that preterm birth and severe asphyxia had a great impact on the early-onset thrombosis in infants without *PROC* mutation(s). Considering that <20% levels of plasma PC activity lead to hypercoagulability, the delivery stresses may reduce the subnormal baseline PC levels and easily raise the chance of thromboembolism in the newborn infants with heterozygous *PROC* mutation.

The present study showed that six variables (purpura fulminans, number of manifestation, gestational age, Apgar score at 5 min, PC activity levels and PC/PS-activity ratio) were indicative of having *PROC* mutation(s). Infants with the mutation(s) developed more severe symptoms and had fewer acquired prothrombotic triggers including premature birth and neonatal asphyxia than those without mutation (Table 1). We have established two formulas consisting of these variables to discriminate mutation carriers from noncarriers with high sensitivities (Formula A; 93%, B; 87%). Only one patient undifferentiated by Formula A had heterozygous mutations including the variant K193del. This mutation has been recently identified as the most common variant in Chinese patients with thromboembolism, although the amidolytic PC activity based screening system does not discriminate the variant carrier from noncarrier [27]. In our Japanese registry, the variant allele of K193del has been occasionally found in patients with late-onset or asymptomatic *PROC* biallelic mutants [15].

One of the limitations in this report was that this screening may be applied only for Japanese patients, because of the ethnic difference. The number of control patients without PROC mutation was small. The other limitation was that the genetic analysis for *PROS1* was not completed in patients with neonatal-onset PC deficiency whose PS activity levels were not depressed (Table 1). In addition, none of the healthy neonates underwent the genetic analysis for PC and PS (Fig. 1). Currently healthy neonates with low-PC activity may have an increased thrombotic risk according to the kind and magnitude of triggers, which need a prospective study on the prolonged observation period. Finally, clinical data were collected in the combination of prospective cohort and retrospective studies because of the rare disease.

## Conclusion

A small group of healthy newborns showed critically low levels (<20%) of plasma PC but not PS activity during the early neonatal period. On the other hand, not all patients with neonatal-onset PC deficiency were demonstrated to have *PROC* mutation as shown in adult patients. Therefore, an established formula including two significant variables of PC activity and PC/PS-activity ratio was required to discriminate patients with *PROC* mutation from those without it. Further prospective studies are required to validate these findings for the early diagnosis and treatment of neonatal-onset PC deficiency.

## Electronic supplementary material

The genotypes of patients with neonatal-onset protein C (PC)-deficiency in the Japanese registry
