# Supplementary material for: Diagnostic challenge of the newborn patients with heritable protein C deficiency
Source: J Perinatol. 2018 Oct 23;39(2):212–9. doi: 10.1038/s41372-018-0262-0 (PMC6760599; doi:10.1038/s41372-018-0262-0)
Supplement: Supplementary file 1 — The genotypes of patients with neonatal-onset protein C (PC)-deficiency in the Japanese registry [file 41372_2018_262_MOESM1_ESM.doc]

**Supplementary table** The genotypes of patients with neonatal-onset protein C (PC)-deficiency in the Japanese registry

-------------------------------------------------------------------------------------------------------------------------------------------------------------------------------------------------

Patient Sex Age at onset Diagnosis PC-activity, % Mutation Reference

-------------------------------------------------------------------------------------------------------------------------------------------------------------------------------------------------

*Biallelic (homozygous or compound heterozygous) mutations*

1 F GA33w Hydrocephaly, PF <10 ex9: c.1141G>A, p.V381M [18]

2 F 0 d ICTH, PF 3 **ex9: c.1268delG, p.G423VfsX82^a^** [18]

3 F GA34w ICTH, PF, Ocular bleeding <10 nd [18]

4 M fetus ICTH <10 ex8: c.688-690delCTG, p.L230del/ **ex9: c.1015G>A, p.V339M** Cohort

5 F 0 d ICTH, PF <10 **ex9: c.1015G>A, p.V339M**/ **ex9: c.1268delG, p.G423VfsX82^a^** [20]

7 nd 1 d PF <5 ex3: c.142G>A, p.E48K/ ex3: c.202G>A, p.E68K 　 Cohort

8 M 1 d ICTH, PF 5 **ex3: c.202G>A, p.E68K**/ **ex9: c.1268delG, p.G423VfsX82^a^** [18]

9 M 2 d ICTH, PF, Ocular bleeding <5 nd [18]

10 M 2 d ICTH, PF, Ocular bleeding <5 **ex9: c.1015G>A, p.V339M/** ex9: c.1003C>T, p.Q335X [18]

11 M 2 d PF 　<10 nd 　 Cohort

12 M 2 d PF, Ocular bleeding 24 nd [18]

13 M 3 d ICTH, PF 2 nd [18]

14 F 4 d ICTH, DVT 17 **ex3: c.202G>A, p.E68K**/ **ex9: c.1015G>A, p.V339M** Cohort

15-1 F 6 d ICTH, Ocular bleeding <5 ex8: c.793C>T, p.L265F/ ex9: c.1266G>C, p.W422C Cohort

15-2 F 6 d ICTH, Ocular bleeding <5 ex8: c.793C>T, p.L265F/ ex9: c.1266G>C, p.W422C Cohort

16 F 13 d ICTH, PF <5 ex4: c.164delT, p.L55RfsX6/ ex9: c.811C>T, p.R271W [17]

17 nd neonate ICTH, PF <5 nd Cohort

18 nd neonate ICTH <5 nd Cohort

*Monoallelic (heterozygous) mutations*

1 M GA28w Hydrocephaly 21 **ex7: c.671-673del AAG, K193del** Cohort

2 F 0 d　　 ICTH, Ocular bleeding <10　　　 nd [18]

3 nd 0 d ICTH 17 ex9: c.970G>A,p.G324S Cohort

4 M 0 d ICTH, PF <10 nd [18]

5 M 0 d ICTH, PF 20 nd [18]

6 M 5 d ICTH 31 nd [18]

7 F 6 d ICTH 19 nd [18]

8 F 10 d ICTH <10 nd Cohort

9 F 16 d ICTH 17 nd [18]

----------------------------------------------------------------------------------------------------------------------------------------------------------------------------------------------

The bold (recurrent) and underlined mutations are the major 5 mutations in Japanese PC deﬁciency, including PC-Nagoya (a) [15].

ICTH, intracranial thrombosis and hemorrhage; PF, purpura fulminans; DVT, deep vein thrombosis; GA, gestational age; M, male; F, female; w, week; d, day; ex, exon; nd, not described/determined.
